# Course, Moderators, and Predictors of Acute Coronary Syndrome-Induced Post-traumatic Stress: A Secondary Analysis From the Myocardial Infarction-Stress Prevention Intervention Randomized Controlled Trial

**DOI:** 10.3389/fpsyt.2021.621284

**Published:** 2021-05-14

**Authors:** Roland von Känel, Rebecca E. Meister-Langraf, Jürgen Barth, Ulrich Schnyder, Aju P. Pazhenkottil, Katharina Ledermann, Jean-Paul Schmid, Hansjörg Znoj, Claudia Herbert, Mary Princip

**Affiliations:** ^1^Department of Consultation-Liaison Psychiatry and Psychosomatic Medicine, University Hospital Zurich, University of Zurich, Zurich, Switzerland; ^2^Clienia Schlössli AG, Oetwil am See, Switzerland; ^3^Complementary and Integrative Medicine, University Hospital Zurich, University of Zurich, Zurich, Switzerland; ^4^Medical Faculty, University of Zurich, Zurich, Switzerland; ^5^Department of Cardiology, University Hospital Zurich, University of Zurich, Zurich, Switzerland; ^6^Cardiac Imaging, Department of Nuclear Medicine, University Hospital Zurich, University of Zurich, Zurich, Switzerland; ^7^Department of Clinical and Health Psychology, University of Fribourg, Fribourg, Switzerland; ^8^Department of Cardiology, Clinic Barmelweid, Barmelweid, Switzerland; ^9^Department of Clinical Psychology and Psychotherapy, University of Bern, Bern, Switzerland; ^10^The Oxford Development Centre Ltd, Witney, United Kingdom

**Keywords:** acute coronary syndrome, emergency psychiatry, post-traumatic stress, prevention, psychological stress, risk factor, trauma stress

## Abstract

Acute coronary syndromes (ACS) induce post-traumatic stress symptoms (PTSS) in one out of eight patients. Effects of preventive interventions, the course and potential moderators of ACS-induced PTSS are vastly understudied. This study explored whether a preventive behavioral intervention leads to a decrease in myocardial infarction (MI)-induced PTSS between two follow-up assessments. Sociodemographic, clinical and psychological factors were additionally tested as both moderators of change over time in PTSS and predictors of PTSS across two follow-ups. Within 48 h after reaching stable circulatory conditions, 104 patients with MI were randomized to a 45-min one-session intervention of either trauma-focused counseling or stress counseling (active control). Sociodemographic, clinical, and psychological data were collected at baseline, and PTSS were assessed with the Clinician-Administered Post-traumatic Stress Disorder Scale 3 and 12 months post-MI. PTSS severity showed no change over time from 3 to 12 months post-MI, either in all patients or through the intervention [mean group difference for total PTSS = 1.6 (95% CI −1.8, 4.9), re-experiencing symptoms = 0.8 (95% CI −0.7, 2.2), avoidance/numbing symptoms = 0.1 (95% CI −1.6, 1.7) and hyperarousal symptoms = 0.6 (95% CI −0.9, 2.1)]. Patients receiving one preventive session of trauma-focused counseling showed a decrease from 3 to 12 months post-MI in avoidance symptoms with higher age (*p* = 0.011) and direct associations of clinical burden indices with total PTSS across both follow-ups (*p*'s ≤ 0.043; interaction effects). Regardless of the intervention, decreases in re-experiencing, avoidance and hyperarousal symptoms from 3 to 12 months post-MI occurred, respectively, in men (*p* = 0.006), participants with low education (*p* = 0.014) and with more acute stress symptoms (*p* = 0.021). Peritraumatic distress (*p* = 0.004) and lifetime depression (*p* = 0.038) predicted total PTSS across both follow-ups. We conclude that PTSS were persistent in the first year after MI and not prevented by an early one-session intervention. A preventive one-session intervention of trauma-focused counseling may be inappropriate for certain subgroups of patients, although this observation needs confirmation. As predictors of the development and persistence of PTSS, sociodemographic and psychological factors could help to identify high-risk patients yet at hospital admission.

## Introduction

Post-traumatic stress disorder (PTSD) is a mental disorder which may develop in individuals who have been exposed to a traumatic event, including experiences involving threatened death or serious injury (1). It is increasingly acknowledged, both conceptually and empirically, that life-threatening physical diseases like heart disease may induce PTSD (2). Specifically, meta-analytic data suggest that acute coronary syndromes (ACS), including acute myocardial infarction (MI), induce PTSD in 4% and clinically significant post-traumatic stress symptoms (PTSS) in 12% of patients (3). Such symptoms include re-experiencing aspects of the MI in thoughts or dreams, avoidance of MI-related activities and situations, and hyperarousal (4).

ACS-induced PTSS are of clinical relevance since they are associated with classic cardiovascular risk factors, unhealthy lifestyle behaviors and poor adherence with cardiac therapy (5). Moreover, ACS-induced PTSS were shown to double the risk of ACS recurrence or mortality, after adjustment for demographic, clinical and psychosocial risk factors, including depression (3, 5), but individual PTSS clusters may differ in terms of risk (6). One reason for their adverse health effects could be that PTSS are often persistent (7–10) or even increase (11) in the first to third year after ACS. However, studies on the trajectory of PTSS, particularly of individual PTSS clusters (12), are scarce and used self-rating questionnaires instead of a gold standard clinical evaluation (6–11). The only prospective study to date applying a clinical interview showed that two thirds of patients with PTSD diagnosed 5 months after MI still qualified for the diagnosis 2 years later (13). Moreover, numerous sociodemographic, clinical and psychological risk factors have been identified as predictors of ACS-induced PTSS (2), but whether they influence the course of PTSS is largely unknown. Such knowledge could inform targeted interventions for subgroups at risk.

Two modalities of intervention could favorably influence the course of ACS-induced PTSS. The first one is to intervene in patients with established ACS-induced PTSS, using for instance psychotherapy or pharmacotherapy, but so far, there is no published randomized controlled trial (RCT) (2, 5). The second one is to prevent the development of PTSS in ACS patients experiencing emergency department threat perception, with for example a psychological first aid approach (5). Regarding the latter quest, we recently conducted the Myocardial Infarction-Stress PRevention INTervention (MI-SPRINT) RCT, the first early behavioral intervention trial in acute MI patients (14). Our preventive one-session intervention was based on principles for psychological first aid approaches characterized by empathic assessment of the current, accurate needs of ACS survivors (15). With this approach, patients are delivered pragmatic psychological support, including information about the possible occurrence of (post-traumatic) stress reactions and symptoms, coping strategies for self-guided help and how to seek for social support and professional help (15). To be beneficial, early interventions should target trauma survivors who have a high risk of developing significant PTSS based on known predictors (16) and trauma-focused (17). Therefore, we recruited patients with high peritraumatic distress during MI, defined as moderate-to-severe fear of dying, helplessness and pain, all of which have been associated with ACS-induced PTSS in previous studies (18–20). Moreover, we developed a single session intervention, targeting the prevention of potentially emerging ACS-induced PTSS after discharge, which could pragmatically be delivered in a busy acute cardiology setting within 48 h of hospital referral for acute coronary intervention (14). Both intention-to-treat and completer analyses showed, however, that early delivered trauma-focused counseling was no better than stress counseling (active control) in preventing interviewer-rated PTSS 3 months (21) and 12 months (to be published elsewhere) post-MI.

Here, we sought to gain new insights into the development, course and response to intervention of the total severity of PTSS and individual clusters of re-experiencing, avoidance/numbing and hyperarousal symptoms due to MI. For this purpose, we drew on data from patients who completed both the 3- and 12-month interview in the MI-SPRINT trial. As these secondary analyses were not pre-planned in the original study protocol (14), they are exploratory and performed without a priori hypotheses, but clearly intended to generate hypotheses for urgently needed future trials in this field (2, 5).

As a novelty, our primary aim was to explore whether a one-session early behavioral intervention, i.e., delivered at hospital admission, would influence the course over time in MI-induced PTSS from 3 to 12 months post-MI. We assumed this might be the case because there is literature suggesting that early interventions, for instance after sexual assault, reduce PTSD severity to a greater extent than standard care at 2 to 12 months follow-up, but not before (22). One explanation could be that PTSS are likely to regress naturally during the first 3 months after a potentially traumatic event. Also new, our secondary aim was to explore nine sociodemographic, clinical, and psychological factors as potential moderators of an intervention effect on both the change over time in PTSS and the severity of PTSS across both follow-up assessments. This to find out which patients might benefit the most of either intervention in the first year post-MI. Our third aim was to explore sociodemographic, clinical, and psychological factors as moderators and predictors of PTSS, independent of an intervention effect, to characterize patients at risk for the development of PTSS over time and persistent PTSS across two follow-up assessments. The novelty of our third aim was the use of a clinician-rated interview to assess PTSS clusters added to total PTSS severity, all of which were assessed at two time points in the first year after MI.

## Materials and Methods

### Study Participants and Design

This is a secondary analysis of data from 104 participants in the MI-SPRINT RCT who completed both the 3 and 12- month assessments of interviewer-rated PTSS. The trial was conducted in a tertiary university hospital in Switzerland between 1/2013 and 12/2015 and registered under ClinicalTrials.gov (NCT01781247). The study protocol was previously published (14) and approved by the ethics committee of the State of Bern. All patients provided written informed consent.

Consecutive patients with verified acute ST-elevation MI (STEMI) or non-STEMI, referred for acute coronary care intervention, were assessed for eligibility and invited to participate in the MI-SPRINT trial. Inclusion criteria were age 18 years or older, stable circulatory conditions and a subjective “high level of peritraumatic distress.” The latter was assessed with numeric rating scales (range 0–10) for “pain intensity (during MI),” “fear of dying (until admission to the coronary care unit)” and “being worried and feeling helpless (when being told about having MI).” High peritraumatic distress was defined by a score of at least “5” for pain plus at least “5” for fear of dying and/or helplessness. Exclusion criteria were emergency coronary artery bypass grafting, severe comorbid diseases, limited orientation, current severe clinical depression (based on the cardiologist's medical history), suicidal ideations in the past 2 weeks, insufficient knowledge of German and participation in another RCT. Patients with cognitive disturbances were also excluded using a two-step procedure. First, if a patient was not fully oriented to situation, person, and place, the study therapist excluded the patient right away. Second, if patients were fully oriented, the study therapist assessed cognitive impairment with an adapted short version of the Mini-Mental State Examination and excluded patients with a score <7 (maximum score is 9) (23).

### Randomization and Masking

As soon as a patient was admitted, the research staff called the study center, where an independent person randomly assigned the participants to either one session of trauma-focused counseling (minimal behavioral intervention group) or one session of stress-focused counseling (control intervention). The randomization list for group allocation was computer-generated with Research Randomizer (www.randomizer.org) and was only accessible to researchers after the end of the entire study.

### Intervention

Both counseling sessions were active face-to-face preventive one-session interventions of the same duration and care, and conducted by the bedside of the coronary care unit within 48 h after patients had reached stable circulatory conditions. Counselors were doctoral students in psychology and medicine, all trained and supervised by senior clinical psychotherapists with degrees in psychology or psychiatry. Each intervention consisted of 45-min counseling plus delivery of an information booklet. Text and figures of the two booklets were specific to each mode of intervention, served as the basis for interacting with patients during the counseling session and to elaborate on certain topics, and for further self-guided help after hospital discharge. Both booklets are available as online material of our previous publication (21). Initially, the counselor dealt with a patient's most immediate concern, before moving on to key issues like “normalization of stress reactions.” For instance, when asking about previous stressful situations (as part of the stress counseling session) and the patient mentioned a difficult situation at work, the discussion was on stress at work. Other forms of stress in life were covered in the session, but the patient could look this up in the information booklet later on.

Trauma-focused counseling applied an educational approach, targeting individual patients' resources and cognitive (re)structuring to prevent any MI-induced traumatic reactions that might occur in the weeks to come. The concept of psychological trauma and PTSD was introduced, including the possibility that MI might induce PTSS. Stress counseling comprised information about the general role of psychosocial stress in cardiac disease and how to use this information in re-building a life after MI, but any trauma-related terminology was strictly avoided. Counseling sessions could be interrupted for medical reasons and later resumed or post-poned on short notice. Pertinent examples and topics, which could specifically be addressed in either 45-min session were for trauma-focused counseling: What is a trauma? What is PTSD in general and related to MI in particular? Why can acute MI be a potentially traumatic event? How do patients cope with and adjust to MI? Which are the expected reactions to a traumatically experienced MI? Why do not all patients react in the same way to MI? How can one cope with traumatic reactions if they occur during follow-up? How can one get professional help? For the stress counseling session, potentially discussed topics were: What is psychosocial stress and when can it become dangerous for the heart? Why do not all patients react the same way to psychosocial stress? Which are the psychosocial stressors with a potential influence on cardiovascular health and prognosis after MI? How can psychosocial stress affect a healthy life style, therapy adherence and cardiovascular biology? How can psychosocial stress be reduced? A more detailed description of the sessions' content can be found elsewhere (14, 21).

### Baseline Measures

All included patients underwent a structured medical history and psychometric assessment before the one-session intervention, except if the sequence had to be changed for medical or logistical reasons. Additional information was taken from patient files. Educational level was categorized as high (university graduation, including applied sciences/high school graduation/matura), medium (apprenticeship or vocational school) or low (lower than apprenticeship or vocational school) (24). Clinical burden was assessed with the Global Registry of Acute Coronary Events (GRACE) risk score (25) and the Charlson comorbidity index (26). The GRACE score considers eight variables (cardiac arrest at hospital admission, ST-segment deviation, elevated cardiac enzymes, age, heart rate, systolic blood pressure, creatinine, Killip class) and is a robust predictor of the cumulative risk of death or recurrent MI from admission to 6 months after discharge (25). On the basis of a Charlson comorbidity index of “1,” “2,” and “≥3,” 10-year mortality risk was classified as low, medium or high, respectively (26).

Peritraumatic distress during acute MI was assessed with three items using numeric rating scales (range 0–10) as described above (27). Individual scores of pain intensity, fear of dying and helplessness during MI were summed and the total divided by three to obtain a total severity score (range 3.33–10). The German version of the 19-item self-rating Acute Stress Disorder Scale was used to assess acute stress disorder symptoms (ASDS), which include symptoms of dissociation, re-experiencing, avoidance and arousal (28). Each item is rated on a 5-point Likert scale (0 = “not at all,” 4 = “extremely”) yielding a total severity score ranging from 0 to 76. In patients who completed all 19 items (see Statistical analysis section for the number and handling of missing values), Cronbach's α for the scale was 0.82, indicating good internal consistency. The severity of depressive symptoms was assessed with the 13-item cognitive/affective subscale of the Beck Depression Inventory (total score 0–39) (29). In patients who completed all 13 items, Cronbach's α for the scale was 0.66, indicating moderate internal consistency. For information on lifetime depression, patients were asked, “Have you ever had a depression in your life? (yes/no).” A 3-item screening questionnaire was used to identify possible PTSD that had emerged in the 3 months prior to MI. Patients were asked if they had experienced a traumatic event before the current hospitalization [1], which came back in nightmares or thoughts they could not get rid of [2] in the last 3 months [3]; an affirmative response to all three criteria identifies PTSD cases in 97% (30).

### Outcome Measures

At 3 and 12 months post-MI, all patients underwent a clinical assessment of PTSS by interviewers who were blinded to group assignments. Mean group differences in the change over time in PTSS was obtained by subtracting 3-month scores from 12-month scores. To assess PTSS, we used the validated German version of the Clinician-Administered PTSD Scale (CAPS) with reference to Diagnostic and Statistical Manual for Mental Disorders (DSM)-IV criteria (31), in official use at the time we planned the trial. With respect to the preceding month, the interviewer rated the frequency and intensity of each of the 17 PTSS between 0 (“never”) and 4 (“almost always”), yielding a total PTSS severity score (range 0–136) and individual scores for the five re-experiencing symptoms (range 0–40), seven avoidance/numbing symptoms (range 0–56) and five hyperarousal symptoms (range 0–40). The minimal clinically important difference for the CAPS total severity score is 10 points (32). A CAPS total severity score of 20 and higher has been proposed as a threshold to indicate clinically significant PTSS (33). For all scales, Cronbach's α was higher at the 3-month than at the 12-month assessment. Internal consistency was good for the CAPS total severity scale (0.81, 0.72), moderate for the re-experiencing (0.71, 0.63) and avoidance/numbing (0.67, 0.57) scales, but poor for the hyperarousal scale (0.48, 0.32).

### Statistical Analysis

There was no power analysis for the exploratory analyses in this study. However, to detect a statistically and clinically meaningful group difference in PTSS 3 months post-MI (primary outcome of the MI-SPRINT trial), a power analysis yielded a sample size of 194 patients per group (14). As previously reported, the target sample size was not achieved, mainly owing to the large number of early hospital discharges resulting from legal changes in Switzerland's health care system, which became effective during the course of the trial (21).

Data were analyzed using SPSS 25.0 for Windows (SPSS Inc., Chicago, IL) with a two-sided level of significance of *p* < 0.05. Multiple imputation (k = 5) was used to impute missing moderator/predictor data (previous MI, specification of index MI, education and lifetime depression missing 1 each; Grace score 5 missings, PTSD screen 8 missings, ASDS and depressive symptoms 17 missings each). Due to a non-normal distribution, CAPS scores were log_10_ transformed and GRACE scores were square root transformed before analysis. Independent samples *t*-test and Pearson chi-square test were used to compare intervention groups on baseline sociodemographic, clinical and psychological variables.

Repeated measures (RM) analysis of (co)variance [AN(C)OVA] was used to test for within-subjects changes over time (from 3 to 12 months post-MI) in the CAPS total severity score and each PTSS cluster separately with “group” (trauma-focused counseling vs. stress counseling) as the between-subjects factor (i.e., 4 exploratory tests for this analysis). For instance, a significant time-by-group interaction would mean that there is a difference in the change over time in the CAPS total severity score between the two counseling groups. Additional adjustment was made for sociodemographic (age, sex, education category), clinical (GRACE score, category of Charlson comorbidity index) and psychological factors (peritraumatic distress, ASDS, lifetime depression, admission PTSD screen). We selected these variables a priori based on the literature on risk factors of ACS-induced PTSS (2, 34), allowing a maximum of nine covariates or predictor variables, respectively, along with group in the equation to guard against model overfitting. We gave preference to lifetime depression over cognitive depressive symptoms at admission to be analyzed as a covariate or predictor variable because patients with current severe depression were excluded for the study, which likely lowered depressive symptomatology in our sample compared with a natural sample.

Three-way interactions were then tested to explore whether these nine sociodemographic, clinical and psychological factors would moderate the response to the intervention of the CAPS total severity score and each PTSS cluster (i.e., 4 × 9 = 36 exploratory tests for this analysis). For instance, to explore whether age is an independent moderator of an intervention effect on changes over time in the CAPS total severity score, we applied RM ANCOVA with “time” as the within-subjects factor (2 time points), and with the age-by-group interaction, age (main effect), group (main effect) and the remaining eight sociodemographic, clinical and psychological variables all entered as covariates. Besides a time-by-age-by-group three-way interaction, the model output also generates an age-by-group two-way interaction with reference to a between-subjects effect. If significant, this would mean that the correlation between age and the CAPS total severity score across the 3- and 12-month follow-up scores is different between the two counseling groups.

Lastly, another set of two-way interactions were tested to explore whether the above nine sociodemographic, clinical and psychological factors would moderate the change over time in the CAPS total severity score and each PTSS cluster separately independent of an intervention effect (i.e., 4 exploratory tests for this analysis). For this purpose we applied RM ANCOVA with “time” as the within-subjects factor (2 time points), and entered all nine predictor variables along with “group” in one block as covariates. For instance, a significant time-by-age two-way interaction for the CAPS total severity score would mean that there is a significant correlation between age and changes over time from 3 to 12 months post-MI in the CAPS total severity score, adjusted for the other covariates in the model. Besides a time-by-age two-way interaction, the model output also generates between-subjects effects. If significant, this would mean that there is a significant correlation between age and the CAPS total severity score across the 3- and 12- month follow-up scores.

Because all analyses were exploratory, with the aim to generate hypotheses for future studies, we did not adjust *p*-values for multiple comparisons. With totally 44 exploratory tests performed, and an alpha level of 0.05, the family wise error rate was 1–(1–0.5)^44^ = 0.895, indicating that the probability of a cumulative type I error in this paper was almost 90%. Effect sizes were expressed as partial eta-squared (η^2^) with 0.01, 0.06, and 0.14 indicating small, medium and large effects, respectively. Cook's distance indicated no influential outliers and variance inflation factors indicated no concern for multicollinearity in the set of predictor variables.

## Results

### Recruitment and Baseline Characteristics of Study Participants

**Figure 1:**
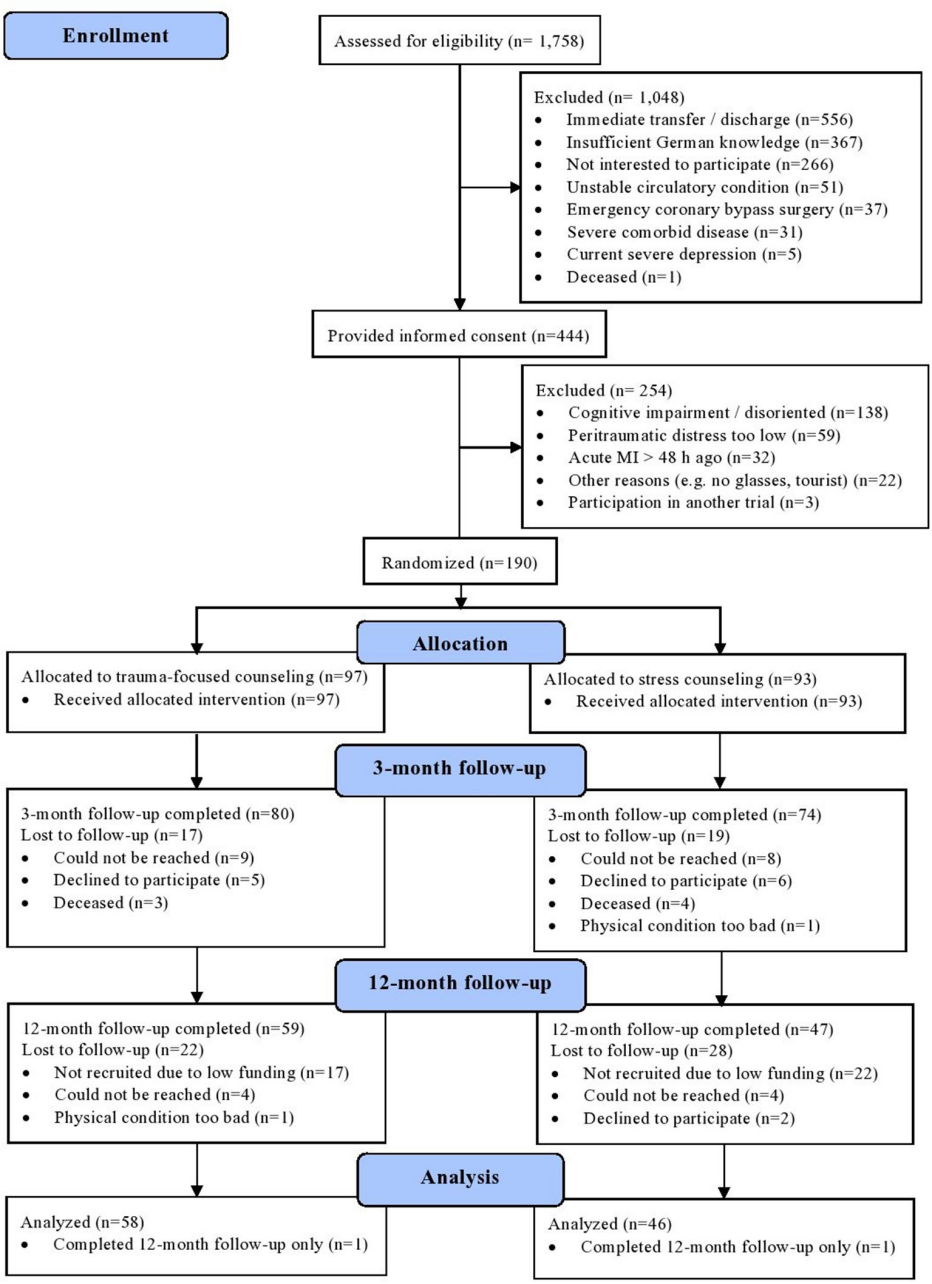
CONSORT flow diagram for the completers (*n* = 104) of the MI-SPRINT trial.

Figure 1 shows the flow of participants through each stage of the MI-SPRINT trial. Of 190 patients who were randomized to either trauma-focused counseling (*n* = 97) or stress counseling (*n* = 93), 154 and 106 completed the CAPS interview 3 and 12 months post-MI, respectively. Since two participants only underwent the 12-month interview, we report data of 104 completers of both CAPS interviews. Of these, 58 had been randomized to trauma-focused counseling and 46 to stress counseling.

Table 1 shows the sociodemographic, clinical and psychological characteristics of the 104 participants per allocation group. Compared with participants in the stress counseling group, those in the trauma-focused counseling group reported significantly more severe peritraumatic distress, mainly due to greater fear of dying. In the entire sample, the median GRACE score corresponded to a mortality risk of 5% (range 1–40) and a median Charlson comorbidity index of 1 (range 1–9) indicated low comorbidity.

**Table 1:** Baseline characteristics of the 104 study participants.

	**Trauma-focused counseling (*n* = 58)**	**Stress counseling (*n* = 46)**	***P*-value for difference**
Age (years)	59.3 ± 10.2	59.2 ± 8.7	0.928
Male sex, *n* (%)	47 (81.0)	39 (84.8)	0.795
Educational level			0.216
High, *n* (%)	14 (24.1)	5 (10.9)	
Medium, *n* (%)	38 (65.5)	36 (78.2)	
Low, *n* (%)	6 (10.4)	5 (10.9)	
ST-elevation MI, *n* (%)	40 (69.0)	32 (69.6)	1.000
GRACE score	103 ± 26	106 ± 25	0.581
Previous MI, *n* (%)	3 (5.2)	6 (13.0)	0.156
Charlson comorbidity index			0.449
High, *n* (%)	14 (24.1)	8 (17.4)	
Medium, *n* (%)	13 (22.4)	15 (32.6)	
Low, *n* (%)	31 (53.5)	23 (50.0)	
Pain intensity (NRS)	8.23 ± 1.54	7.85 ± 1.80	0.243
Fear of dying (NRS)	6.03 ± 2.61	4.33 ± 2.85	0.002
Helplessness (NRS)	5.61 ± 2.86	5.50 ± 2.49	0.834
Peritraumatic distress	6.63 ± 1.25	5.89 ± 1.31	0.004
Acute stress disorder symptoms	17.8 ± 11.3	15.0 ± 8.8	0.168
PTSD screen positive, *n* (%)	7 (12.1)	1 (2.2)	0.061
Lifetime depression, *n* (%)	13 (22.4)	14 (30.4)	0.241
Cognitive depressive symptoms	2.76 ± 2.37	2.85 ± 2.90	0.865

*Continuous variables are given as mean values with standard deviation. GRACE, Global Registry of Acute Coronary Events; MI, myocardial infarction; NRS, numeric rating scale; PTSD, post-traumatic stress disorder*.

### Post-traumatic Stress Symptoms and Intervention Effects

**Figure 2:**
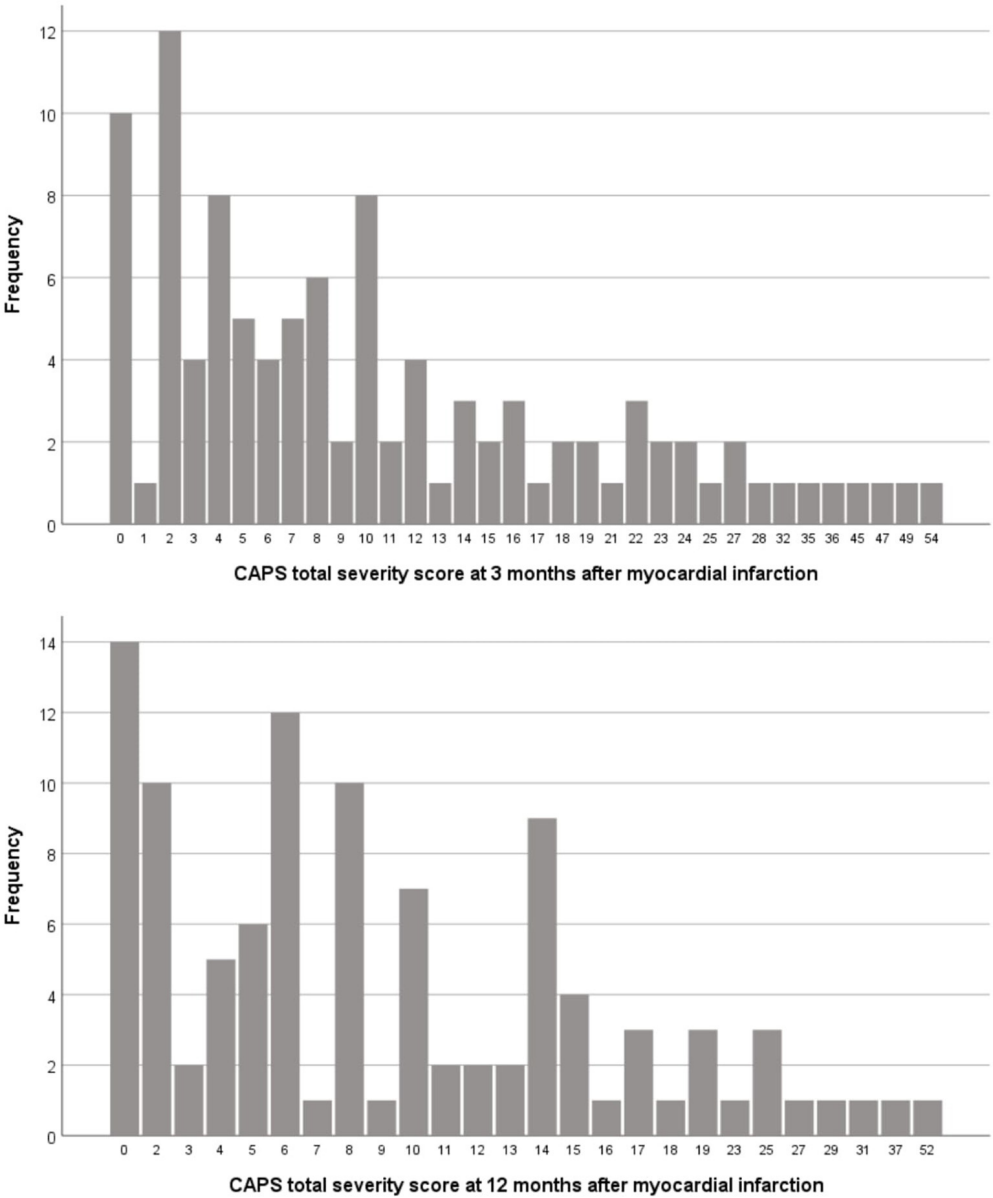
Distribution of CAPS total severity scores at the two follow-up assessments. CAPS, Clinician-Administered Post-traumatic Stress Disorder Scale.

Figure 2 displays the distribution of the CAPS total severity scores at both follow-up investigations. Clinically significant PTSS were present in 19 (18.3%) patients 3 months post-MI and in 9 (8.7%) patients 12 months post-MI. Table 2 shows the severity of PTSS in each intervention group 3 and 12 months post-MI along with the results of the repeated measures ANOVA. Compared with stress counseling, trauma-focused counseling resulted in higher CAPS scores. Mean group differences [12-month CAPS score−3-month CAPS score (original units)] were 1.6 [95% confidence interval (CI) −1.8, 4.9] for the CAPS total severity score, 0.8 (95% CI −0.7, 2.2) for re-experiencing symptoms, 0.1 (95% CI −1.6, 1.7) for avoidance/numbing symptoms and 0.6 (95% CI −0.9, 2.1) for hyperarousal symptoms. However, these differences were not significant (i.e., no significant time-by-group interaction for any CAPS score). Moreover, non-significant time effects indicated that the severity of PTSS did not change over time across all participants, and non-significant group effects indicated equally severe 3- and 12-month PTSS. All time-by-group interactions, time effects and group effects remained non-significant with adjustment for age, sex, education, GRACE score, Charlson comorbidity index, peritraumatic distress, ASDS, lifetime depression, and admission PTSD screen.

**Table 2:** Post-traumatic stress symptoms 3 and 12 months after myocardial infarction.

**CAPS score**	**Intervention group**	**3 months**	**12 months**	***P***
Total severity	Trauma-focused counseling	11.6 (8.7–14.5)	10.5 (8.3–12.7)	
	Stress counseling	11.0 (7.5–14.5)	8.3 (5.6–11.0)	
	Time effect			0.153
	Time × group effect			0.224
	Group effect			0.155
Re-experiencing	Trauma-focused counseling	2.7 (1.7–3.7)	2.3 (1.6–3.0)	
	Stress counseling	2.9 (1.5–4.4)	1.8 (0.7–2.8)	
	Time effect			0.108
	Time × group effect			0.134
	Group effect			0.322
Avoidance	Trauma-focused counseling	3.9 (2.4–5.3)	3.4 (2.3–4.6)	
	Stress counseling	2.8 (1.8–3.9)	2.3 (1.2–3.5)	
	Time effect			0.153
	Time × group effect			0.323
	Group effect			0.182
Hyperarousal	Trauma-focused counseling	5.0 (3.8–6.1)	4.5 (3.5–5.5)	
	Stress counseling	5.2 (3.7–6.7)	4.2 (3.1–5.3)	
	Time effect			0.282
	Time × group effect			0.817
	Group effect			0.799

*Data are mean values with 95% confidence interval. CAPS, Clinical-Administered Post-traumatic Stress Disorder Scale*.

### Predictors of a Response to Post-traumatic Stress Prevention Intervention

**Figure 3:**
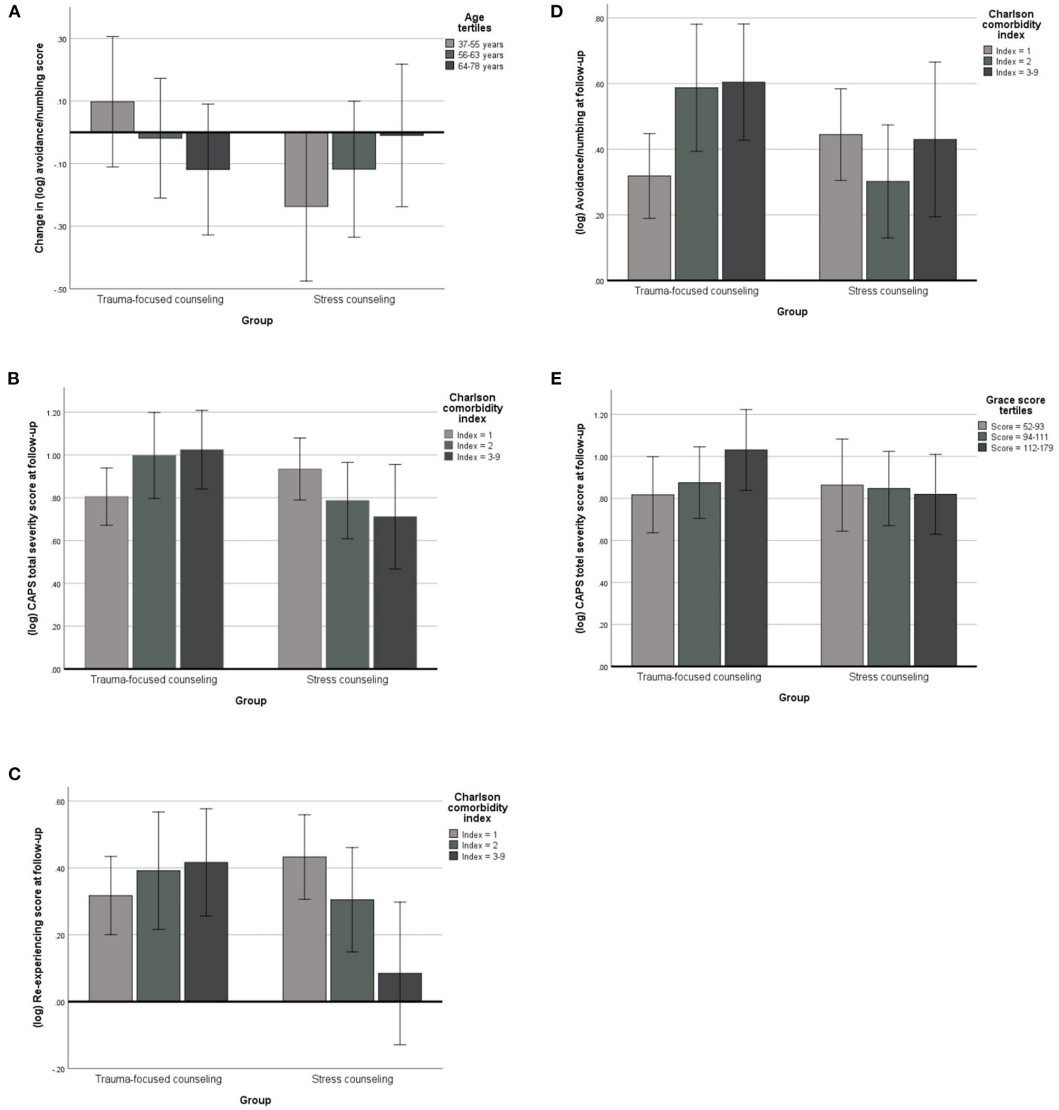
Moderators of intervention effects on post-traumatic stress symptoms. CAPS, Clinician-Administered Post-traumatic Stress Disorder Scale; CAPS scores are expressed as base-10 logarithmically (log) transformed values, which were used in regression models due to a non-normal distribution of original CAPS scores. The bar charts (mean values with 95% confidence interval) illustrate the independent associations between significant moderators of an intervention effect on the change over time from 3- to 12 months in CAPS scores **(A)** and on CAPS scores across both these follow-ups **(B–E)**. Adjustments were made for the other variables in the model. These were age, sex, education, Charlson comorbidity index, GRACE score, peritraumatic distress, acute stress disorder symptoms, lifetime depression, and admission post-traumatic stress disorder screen. The mean log value of a CAPS score (y-axis) can be converted back to a base-10 a log mean of 0.1 corresponds to (≡) a geometric mean of 1.3; 0.2 ≡ 1.6, 0.3 ≡ 2.0, 0.4 ≡ 2.5, 0.5 ≡ 3.2, 0.6 ≡ 4.0, 0.7 ≡ 5.0, 0.8 ≡ 6.3, 0.9 ≡ 7.9, 1.0 ≡ 10.0, 1.1 ≡ 12.6, and 1.2 ≡ 15.9.

With the following tests, we explored age, sex, education, GRACE score, Charlson comorbidity index, peritraumatic distress, ASDS, lifetime depression, and admission PTSD screen each as potential moderators of intervention effects on changes over time from 3 to 12 months post-MI in PTSS and on PTSS across both follow-up assessments. To yield independent effects, all interactions were simultaneously adjusted for age, sex, education, GRACE score, Charlson comorbidity index, peritraumatic distress, ASDS, lifetime depression and admission PTSD screen. The significant results of these analyses are illustrated in Figure 3.

#### Moderators of Intervention Effects on Changes Over Time in PTSS

There was a significant *time-by-age-by-group interaction* for avoidance/numbing symptoms (*F* = 6.80, *p* = 0.011; partial η^2^ = 0.069). Figure 3A illustrates that older participants in the trauma-focused counseling group experienced a decrease in avoidance/numbing symptoms over time relative to older participants in the stress counseling group who showed an increase.

#### Moderators of Intervention Effects on PTSS Across Both Follow-Ups

There was a significant *Charlson comorbidity index-by-group interaction* for the CAPS total severity score (*F* = 6.59, *p* = 0.012; partial η^2^ = 0.067) and symptoms of re-experiencing (*F* = 7.71, *p* = 0.007; partial η^2^ = 0.077) and avoidance/numbing (*F* = 4.11, *p* = 0.045; partial η^2^ = 0.043) across both follow-ups. In the trauma-focused counseling group, greater comorbidity was associated with a higher CAPS total severity score (Figure 3B), as well as more re-experiencing (Figure 3C) and avoidance/numbing (Figure 3D) symptoms, whereas in the stress counseling group, greater comorbidity was associated with less severe PTSS. There was also a significant *Grace score-by-group interaction* for the CAPS total severity score (*F* = 4.21, *p* = 0.043; partial η^2^ = 0.044). Figure 3E illustrates that a higher GRACE score was associated with a higher CAPS total severity score in participants in the trauma-focused counseling group, relative to those in the stress counseling group who showed an inverse association between the GRACE score and CAPS total severity score.

### Sociodemographic, Medical, and Psychological Predictors of PTSS

In this last series of tests, we aimed to identify sociodemographic, clinical and psychological factors as independent predictors of changes over time from 3 to 12 months post-MI in PTSS and of PTSS across both follow-up assessments. For this purpose, age, sex, education, GRACE score, Charlson comorbidity index, peritraumatic distress, ASDS, lifetime depression, and admission PTSD screen were entered into the model together, adjusting for group. The significant results of these analyses are presented in Table 3.

**Table 3:** Predictors of post-traumatic stress symptoms.

**Significant predictor**	**Predictors of changes over time in PTSS**			
	**Total severity**	**Re-experiencing**	**Avoidance**	**Hyperarousal**
Female vs. male sex		Points = 2.9 (1.0, 4.8)		
High vs. low education			Points = 4.0 (0.9, 7.0)	
ASDS				B = −0.091 (−0.181, −0.001)
	**Predictors of PTSS across both follow-ups**			
	**Total severity**	**Re-experiencing**	**Avoidance**	**Hyperarousal**
Peritraumatic distress	B = 0.089 (0.029, 0.149)	B = 0.075 (0.023, 0.128)		B = 0.082 (0.029, 0.136)
Lifetime vs. no lifetime depression	Points = 5.9 (1.8, 10.0)			Points = 3.2 (1.6, 4.8)
ASDS			B = 0.008 (0.001, 0.016)	

*ASDS, acute stress disorder symptoms; PTSS, post-traumatic stress symptoms*.

*Data are mean differences [original units] in Clinician-Administered Post-traumatic Stress Disorder Scale score points for categorical predictors and unstandardized coefficients B with 95% confidence interval for continuous predictors, adjusting for the other predictors in the model. These were age, sex, education, Charlson comorbidity index, GRACE score, peritraumatic distress, acute stress disorder symptoms, lifetime depression, admission post-traumatic stress disorder screen, and intervention group*.

#### Predictors of Changes Over Time in PTSS

There was a significant *time-by-sex interaction* for re-experiencing symptoms (*F* = 7.82, *p* = 0.006; partial η^2^ = 0.078). Whereas, male participants showed a decrease, female participants showed an increase over time in severity of re-experiencing symptoms. A significant *time-by-education interaction* emerged for avoidance/numbing symptoms (*F* = 6.21, *p* = 0.014; partial η^2^ = 0.063). Compared to participants with low education, those with high education showed an increase over time in severity of avoidance/numbing symptoms. There was a significant *time-by-ASDS interaction* for hyperarousal symptoms (*F* = 5.48, *p* = 0.021; partial η^2^ = 0.056) such that participants with more severe ASDS showed a greater decrease over time in severity of hyperarousal symptoms.

#### Predictors of PTSD Symptoms at Follow-Up

There was a significant *main effect of peritraumatic distress* for the CAPS total severity score (*F* = 8.60, *p* = 0.004; partial η^2^ = 0.085), symptoms of re-experiencing (*F* = 8.04, *p* = 0.006; partial η^2^ = 0.080) and hyperarousal (*F* = 9.26, *p* = 0.003; partial η^2^ = 0.091) across both follow-ups. Peritraumatic distress was associated with more severe PTSS. For a 5.5-point increase of peritraumatic distress, there was an increase of [original units] 10.1 points (95% CI 2.1, 18.1) in the CAPS total severity score, identical with a clinically important difference (24). There was a significant *main effect of lifetime depression* for the CAPS total severity score (*F* = 4.44, *p* = 0.038; partial η^2^ = 0.046) and hyperarousal symptoms (*F* = 8.60, *p* = 0.004; partial η^2^ = 0.085) across both follow-ups. Participants with lifetime depression had more severe PTSS than those without lifetime depression. Finally, a significant *main effect of ASDS* emerged for avoidance/numbing symptoms (*F* = 4.64, *p* = 0.034; partial η^2^ = 0.048) such that participants with more severe ASDS showed more severe avoidance/numbing symptoms across follow-ups.

## Discussion

### Principal Findings From the Minimal Behavioral Intervention

We previously reported no significant difference in interviewer-rated PTSS, both 3 months (21) and 12 months (to be published elsewhere) after MI, between patients who had received a preventive one-session intervention of trauma-focused vs. stress counseling at hospital admission. Confirming a lack of attrition bias in these previous studies, we obtained the same result in the current study on the 104 completers of both follow-ups. As a novel result, we found that interviewer-rated PTSS did not change between 3 and 12 months post-MI in response to the one-session intervention. Low statistical power is one explanation, but, whatsoever, the mean group difference of 1.6 points for the change over time in the CAPS total severity score was far from the minimal clinically important difference of 10 points (32). However, although not statistically significant, there even seemed to be a pattern of differences that went in the opposite direction of the original hypothesis. For instance, there was a 38% decrease over time in re-experiencing symptoms in the stress counseling group (from 2.9 to 1.8), but only 11% in the trauma-focused counseling group (from 2.7 to 2.4). This difference could still be clinically significant, as intrusive memories of a heart attack can be a very distressing experience. Sociodemographic, clinical and psychological factors did not account for this result. Another explanation for the lack of an intervention effect could be a floor effect, as many of our patients did not endorse much PTSS. Nonetheless, consistent with the results of a meta-analysis (3), 18.3 and 8.7% of patients had clinically significant PTSS 3 and 12 months after MI, respectively.

We also found little evidence that these factors moderated an intervention response of changes over time in PTSS. Of totally nine factors tested, age alone emerged as a significant and independent moderator, with older participants in the trauma-focused counseling group experiencing a reduction in avoidance/numbing symptoms from 3- to 12 months post-MI. In contrast, we found two indices of clinical burden and mortality risk to be moderators of an intervention effect on the severity of PTSS at follow-up. That is, both a higher GRACE score and a higher Charlson comorbidity index were significantly and independently associated with greater severity of total PTSS in patients having received trauma-focused counseling. In this group, the Charlson comorbidity index showed an additional direct association with re-experiencing and avoidance/numbing symptoms. Small-to-moderate effect sizes implied clinical relevance. Mean values for both the GRACE score and the Charlson comorbidity index were very similar to those observed in previous studies on ACS-induced PTSS in patients of the same age as in our study (6, 8, 11).

### Clinical Implications of the Intervention Findings

Patients with more severe ACS and medical comorbidity could develop more severe ACS-induced PTSS in the first year post-MI. One reason for this could be that patients who are aware of their poor physical health and of the prognostic impact of PTSS may pay specific attention to PTSS should these occur during follow-up. As PTSS may increase during the course of treatment before improvement occurs (35), another explanation could be that our assessment captured only the transient increase in PTSS in patients with high clinical burden. Alternatively, patients with more severe ACS and medical comorbidity could benefit from preventive stress counseling and hence less adverse outcome, as ACS-induced PTSS have been associated with poor prognosis (3). However, due to the semi-continuous nature of the applied clinical burden variables (i.e., GRACE score and Charlson comorbity index), recommendations for actual clinical practice cannot readily be made.

In a larger context of ongoing efforts made to design effective early interventions to prevent PTSD in survivors of life-threatening medical events, the MI-SPRINT randomized controlled trial is the first of this kind in patients with ACS (36). We took a pragmatic approach by designing a preventive one-session intervention that could be easily implemented in daily practice of a busy emergency cardiology setting. As stated in the study protocol (14), we were particularly anxious to avoid elements of single-session critical incident stress debriefing, which has been criticized, as it can increase the risk of developing PTSS when applied to individuals who experienced acute psychological trauma (17, 37). However, the results of our previous studies (21, to be published elsewhere) and the results presented here suggest that one single early counseling session is probably insufficient to favorably influence the development and course of clinician-rated PTSS in the average ACS patient. At the same time, these findings could also be a warning about potential iatrogenic effects of a very brief, one-session trauma-focused treatment as it relates to continuous variables, such as age and clinical burden. Results might have yielded a group difference, if we had not chosen an active control intervention or if we had delivered a higher number of counseling sessions, as has been demonstrated for multiple session early psychological interventions for the prevention of PTSS, although not yet in patients with ACS (38). However, in patients who received an implantable cardioverter defibrillator for primary prevention of sudden cardiac death, cognitive behavioral therapy with eight telephone counseling sessions resulted in improvement of self-rated PTSS compared to usual cardiac care after 12 months (39).

### Principal Findings and Implications Irrespective of the Intervention

In analyses adjusted for a preventive one-session intervention effect, we found no significant difference in the severity of total PTSS and PTSS clusters between the 3- and 12-month follow-up assessments. This observation confirms earlier studies suggesting that MI-induced PTSS may persist in the first year after ACS to show a decrease in only a fraction of patients thereafter (6–13). However, in most of these previous studies, researchers did neither apply a clinical interview to rate PTSS nor did they perform analyses in terms of individual PTSS clusters. The tendency of MI-induced PTSS to become chronic together with their prognostic impact make it even more necessary to find effective treatments (2, 5).

There are very few studies on trajectories in ACS-induced PTSS, all using self-assessment tools. One study found that greater pain intensity at admission was associated with delayed recovery of PTSS over 3 years (9). Another study found emergency department threat perception to differentiate patients who developed PTSS during the first year post-MI from those who did not (11). Our study yielded three moderators. Compared to men, women showed an increase in re-experiencing symptoms. This trajectory could put women at risk for future major adverse cardiac events and all-cause mortality, which were previously predicted by re-experiencing symptoms (6). Interestingly, we found an increase in avoidance/numbing symptoms in patients with high education, perhaps because this group was more concerned with the topic than the low-education group with less health literacy, but this remains speculative. Patients with more severe ASDS experienced a greater decrease in hyperarousal symptoms over time, which could reflect regression to the mean.

Psychosocial factors are more clearly predictive for the development of ACS-induced PTSS than objective measures of ACS severity (19, 40–42). We confirmed this literature by identifying lifetime depression history (8, 19) and peritraumatic distress (8, 10, 20, 43) as independent risk factors for total PTSS and some PTSS clusters across two follow-up assessments. An increase from the minimum score of 3.3 on the peritraumatic distress scale to a score of 8.8 was associated with an increase on the CAPS total severity scale of 10 points, indicating a clinically important difference. More severe ASDS predicted more severe avoidance/numbing symptoms, but not total PTSS as in two previous studies, which, however, did not assess individual PTSS clusters (19, 40). As expected, the GRACE score was not an independent predictor of PTSS. Female sex, younger age, and lower education have been reported as risk factors of PTSS in earlier studies, but the literature is not consistent (2), one potential explanation for why sociodemographic variables were not predictors in our study.

### Strengths and Limitations

The clinical assessment of MI-induced PTSS at two time points and the RCT design are strengths of our study. The longitudinal design allowed prospective testing of sociodemographic, medical and psychological variables as moderating or direct independent risk factors of PTSS outcomes beyond mere cross-sectional associations. The fact that we conducted only a single session of trauma-focused counseling limits any conclusions about whether our results were unhelpful for specific subgroups of patients with ACS. By limitation of the sample size, we could not include additional important variables like social support (10) and personality traits (44) in models, and small effects could have failed to reach statistical significance. The group difference in peritraumatic distress pre-intervention makes it difficult to interpret the exploratory ANCOVA findings. We used a non-standardized scale to screen potential participants for peritraumatic stress such as the acute stress disorder scale. This may have influenced the results of our study and the conclusions drawn. To test hypotheses for future studies, we carried out a considerable number of exploratory tests, so there is a high risk of obtaining significant findings by chance. Alpha inflation was indeed huge with a probability of almost 90% for a cumulative type I error. Therefore, it is premature to draw firm conclusions from these analyses. The reliability of the hyperarousal scale was poor, meaning that findings for this symptom cluster must be interpreted with caution. One reason could be that the hyperarousal symptoms of PTSD overlap with those of depression and anxiety disorders, which are common in patients after MI. While the CAPS interview ties re-experiencing and behavioral avoidance symptoms specifically to the traumatic experience of MI, this is not the case for the hyperarousal symptoms. Difficulty concentrating, sleep problems, hypervigilance and irritability are also common symptoms of anxiety and depression, potentially compromising their reliability as specific hyperarousal symptoms of ACS-induced PTSD. Although we excluded patients with cognitive impairment from the study, we did not assess cognitive status at follow-up. Cardiac diseases may be accompanied by worsening of cognitive function over time, which may have affected patients' accurate reporting of PTSS. Whether the results of our study would hold with the new DSM-5 criteria for PTSD is unclear. The challenges of and the need for flexibility in conducting an RCT in an emergency medical environment with unpredictable day-to-day business are obvious and have already been discussed elsewhere (21). The results of our study from a single university center on highly distressed patients may not be transferable to other healthcare settings.

## Conclusions

Drawing on clinical data from a randomized controlled preventive one-session behavioral intervention trial (14), the exploratory analyses of the present study yielded important novel insights into the trajectory, moderators and risk factors of clinician-rated ACS-induced PTSS. As PTSS were lasting and did not respond to a preventive one-session intervention in the average MI patient, the ongoing search for effective early interventions in patients at risk to develop ACS-induced PTSS remains an ultimate need. As preventive one-session interventions may not be sufficiently effective for this purpose, multisession early interventions could be a logic next step to be tested in future trials (38). Already at the time of admission, demographic and psychological factors could help to identify patients at risk for persistent and severe MI-induced PTSS such that early preventive means can be initiated.

## Data Availability Statement

The anonymized data that support the findings of this study are available from the corresponding author upon reasonable request.

## Ethics Statement

The studies involving human participants were reviewed and approved by Ethics committee of the State of Bern (Switzerland). The patients/participants provided their written informed consent to participate in this study.

## Author Contributions

RvK, US, HZ, JB, JPS, and CH conceived and designed the study. RvK performed statistical analysis. RvK, MP, and RM-L handled funding and supervision. RM-L and MP acquired the data. RvK, JB, AP, and US drafted the manuscript. HZ, CH, JPS, MP, and RM-L made critical revision of the manuscript for key intellectual content. All authors approved the final version of the manuscript for submission.

## Conflict of Interest

CH was employed by The Oxford Development Centre Ltd. The remaining authors declare that the research was conducted in the absence of any commercial or financial relationships to The Oxford Development Centre Ltd that could be construed as a potential conflict of interest.
